# Dunglison’s Medical Dictionary

**Published:** 1874-04

**Authors:** 


### Books Reviewed.

---

*A Dictionary of Medical Science; containing a concise explanation of the various subjects, and terms of Anatomy, Physiology, Pathology, Therapeutics, etc., etc., with Accentuation and Etymology of the Terms and the French and other Synomyms.* By Robley Dunglison, M. D., L.L.D. A New Edition Enlarged and Revised. By Richard J. Dunglison, M. D. Philadelphia: Henry C. Lea, 1874. Buffalo: T. Butler & Son.

Dunglison's Dictionary has long been regarded as the standard Medical Lexicon by American physicians In the large mass of medical works which have emenated from American writers, none we think, will reflect more credit upon its author, or upon the profession as a whole than Dunglison's Dictionary.

The careful painstaking work which must have been necessary to produce a work of the magnitude and general excellence of the one under consideration, can never be appreciated by those who use it.

The present edition has been carefully and completely revised under the supervision of the authors son, Dr. Richard J. Dunglison, whose previous training in assisting the author in the preparation of former editions, eminently fitted him for the duty.

The Preface informs us that over six thousand terms not embraced in the former edition, are included in this, making an increase in the size of the volume equivilant to one hundred and sixty pages of the last edition It is useless for us to discuss the comparative merits of a work so well known as this. It stands before the profession confessedly the best medical lexicon in the English language,

